# Rheumatic Diseases Amidst Conflict in Northwest Syria: Unveiling Health Challenges and Implications

**DOI:** 10.1055/s-0044-1786826

**Published:** 2024-05-16

**Authors:** Wasim Zakaria, Yousef Ibrahim

**Affiliations:** 1 Department of Internal Medicine, Syrian Board of Medical Specialties, Idlib, Syria; 2 Department of Rheumatology, Syrian Board of Medical Specialties, Idlib, Syria

**Keywords:** northwest Syria, conflict, rheumatic diseases, rheumatologists, health care system, health care professionals, cost

## Abstract

**Background** The ongoing conflict in Syria has significantly affected the health care system, particularly in the realm of rheumatology. The purpose of this study is to assess the current state of rheumatic diseases in the northwestern region of Syria, where the health care infrastructure has been severely impacted.

**Methods** This was a retrospective study reviewing all patients with rheumatologic conditions seen in internal medicine clinics in northwest Syria between September 2019 and February 2022. Baseline demographic data and diagnoses were collected retrospectively, without any data duplication, from outpatient clinic records. The study also reviewed the availability of investigations and drugs in the northwestern region of Syria.

**Results** We analyzed data from 488 patients (average age: 37.4; 63% female) diagnosed with rheumatic diseases. The most prevalent condition was connective tissue disorders (25.6%), with osteoarthritis (12.1%) and rheumatoid arthritis (8.2%) following. The ongoing conflict has led to a significant shortage of rheumatologists, with only three serving a population of 5.5 million. Furthermore, the conflict has disrupted the provision and quality of rheumatology diagnostic tests, reducing patient accessibility. The dearth of medications and increased costs have compounded the complexity of health care for those with rheumatic diseases.

**Conclusions** This study highlights the urgent need for improved health care services and proposes solutions to address gaps in rheumatic care in northwest Syria.

## Introduction

Armed conflicts have devastating effects that often lead to loss of life, injury, displacement of populations, and destruction of vital infrastructure such as health care systems. The Syrian war is one of the most tragic humanitarian crises of the twenty-first century, having begun in 2011 and now entering its 13th year as of March 2024. It has caused millions of people to be displaced, both inside and outside of Syria, and hundreds of thousands of individuals have lost their lives, become disabled, or gone missing as a result. It is crucial that we continue to work toward finding peaceful solutions to conflicts like these to prevent such human suffering in the future. 1

The Syrian revolution, which was part of the Arab Spring in 2011, turned into the largest refugee crisis of modern times. Millions of Syrians were forced to flee their homes and become refugees in other countries. Additionally, over 6 million people were internally displaced, and the region suffered from massive destruction. This destruction affected the essential infrastructure, the health care system, the social status of the population, and the economy, leading to a growing need for humanitarian aid from the international community. 1 Unfortunately, the international community failed to prevent the destruction of health infrastructure, which caused the collapse of Syria's health care system and left millions of displaced people in dire need of medical assistance. 1

The ongoing conflict and violence in Syria have had a direct impact on the health care facilities and workers in the country. The public health care system has been severely damaged, which has resulted in serious health consequences for the population. 2 These consequences include an increased risk of communicable and noncommunicable diseases, significant challenges related to maternal and child health, trauma caused by the conflict, mental health issues, and a large-scale migration of Syrian health care workers who are seeking to escape the ongoing conflict. 1 3 The prolonged conflict in Syria for over 12 years has impacted the diagnosis and treatment of rheumatic diseases.

There is a shortage of health care professionals specializing in rheumatic diseases worldwide, particularly in low- and middle-income countries. 4 This issue is more severe in areas affected by long-term conflicts. For instance, in Syria, the number of doctors has decreased significantly from 11,305 (0.529 doctors per 1,000 population) in 2010 before the conflict started to 5,889 doctors (0.291 doctors per 1,000 population) in 2018. 5 There has been a significant decrease in the quality of health care services in many areas due to several factors. These factors include economic pressure caused by migration, deliberate targeting of health care workers by combatants, and the weakening of medical education systems. The medical education system is suffering from a mass exodus of teachers and doctors, destroyed infrastructure, limited resources (including the internet), shortages of medicines and laboratory tests, disruption of mobility due to safety concerns, and a shift in focus on health care.

During the Syrian war, there were only 60 rheumatologists available in the country, which equates to 0.26 doctors per 100,000 people. In Aleppo, a neighboring city in northern Syria with a population of 4.8 million, there were only five rheumatologists available, which means there were only 0.1 rheumatologists per 100,000 people. It is important to note that this information was obtained by personal contact given lack of documented and accurate data in Syria

The purpose of this study is to discuss the issue of rheumatic diseases during a 2-year conflict period. It provides an overview of the current health situation regarding rheumatic diseases, outlines the challenges faced by the health system in this area, and suggests solutions to improve rheumatology in conflict-affected areas, such as northwest Syria.

## Methods

This study examined rheumatology patients who visited internal medicine outpatient clinics, which were supervised by one internal medicine physician (corresponding author, W. Z.), in five hospitals located in northwest Syria. This area is currently under the control of nonstate forces and the Turkish government, as it has lost all support from the Syrian government. The study collected baseline demographic data and diagnosis retrospectively, without any duplication of data, between September 2019 and February 2022, from outpatient clinic records. The patients were diagnosed according to the latest criteria of the European League Against Rheumatism and the American College of Rheumatology, which were published on the Web site of the American College of Rheumatology. 6

This article reviews investigations and drugs that are either available or unavailable in the northwestern region of Syria. However, it is important to note that the drugs or investigations may be present in other regions such as areas controlled by the regime or neighboring regions such as Turkey.

The cost of medical investigations and drugs depends on the purchasing power of patients. These costs can be categorized as either affordable or nonaffordable. “Affordable” could be defined as a cost that does not exceed a certain percentage of an individual's income However, some patients may not be able to afford the expenses of their treatment and may have to rely on charitable organizations for financial support. In some cases, patients are forced to take on debt to purchase the necessary medications. The collected data were analyzed using the Statistical Package for Social Sciences (SPSS v.24; SPSS Inc., Chicago, IL, United States). The study was approved by the Institutional Review Board (IRB) at the Idleb Health Directorate. Due to the retrospective nature of the study and the use of an existing, de-identified dataset that was collected without explicit research purposes, the requirement for informed consent was waived by the IRB.

## Results

### Current Health Status Regarding Rheumatic Diseases

#### Demographic Characteristics and Rheumatic Disease Diagnoses of the Study Population

A clinic in northwest Syria treated 488 patients with rheumatic diseases over 2 years (average age, 37.4; 63% female). Connective tissue disorders dominated (25.6%), with rheumatoid arthritis affecting 8.2% and lupus 1.0%. Osteoarthritis (12.1%) and gout (5.3%) were common musculoskeletal diagnoses. Seronegative spondyloarthropathies (5.9%) included axial (3.3%) and peripheral (1.2%) subtypes. Juvenile idiopathic arthritis (3.1%) and Behcet's disease (11.3%) were notable in pediatric and nonmusculoskeletal categories. This diverse patient profile highlights the complexity of rheumatic care in conflict zones. Table 1 provides a brief overview of demographic information and prevalent diagnoses across different categories, with emphasis on the context of a conflict zone.

**Table 1:** Demographic characteristics and rheumatic disease diagnoses of the study population

Disease	Frequency, *n*	Percent	Female, *n* (%)	Male, *n* (%)	Mean age, y
Total	488	100	309 (63.3)	179 (36.7)	37.43
Connective tissue disorders					
Rheumatoid arthritis	125	25.6	102 (81.6)	23 (18.4)	41.78
Systemic lupus erythematosus	40	8.2	38 (95)	2 (5)	31.63
Systemic sclerosis (scleroderma)					
Diffuse cutaneous systemic sclerosis	5	1.0	5 (100)	0 (0)	45.40
Limited cutaneous systemic sclerosis	11	2.3	8 (72.7)	3 (27.3)	32.73
CREST syndrome	6	1.2	4 (66.7)	2 (33.3)	39.50
Raynaud's disease	10	2.0	6 (60)	4 (40)	35.60
Dermatomyositis	6	1.2	3 (50)	3 (50)	29.83
Sjogren's syndrome	6	1.2	6 (100)	0 (0)	42.17
Mixed connective tissue disease	5	1.0	5 (100)	0 (0)	28.40
Antiphospholipid syndrome	4	0.8	4 (100)	0 (0)	31.75
Osteoarthritis and related disorders					
Osteoarthritis	59	12.1	45 (76.3)	14 (23.7)	49.12
Diffuse idiopathic skeletal hyperostosis (DISH)	3	0.6	5 (100)	0 (0)	65.33
Crystal-induced arthropathies					
Gout	26	5.3	7 (26.9)	19 (73.1)	51.58
Seronegative spondyloarthropathies					
Axial spondyloarthropathy	29	5.9	6 (20.7)	23 (79.3)	33.28
Peripheral spondyloarthritis	6	1.2	4 (66.7)	2 (33.3)	29.50
Reactive arthritis	6	1.2	2 (33.3)	4 (66.7)	25.67
IBD-associated arthritis	3	0.6	1 (33.3)	2 (66.7)	25.00
Psoriatic arthritis	3	0.6	2 (66.7)	1 (33.3)	38.00
Fever-associated rheumatic disorders and pediatric disorders					
Adult-onset Still's disease	4	0.8	3 (75)	1 (25)	31.75
Familial Mediterranean fever	11	2.3	4 (36.4)	7 (63.6)	14.45
Juvenile idiopathic arthritis	15	3.1	4 (26.7)	11 (73.3)	10.20
Vasculitides					
Giant cell arteritis	1	0.2	0 (0)	1 (100)	50.00
Polymyalgia rheumatica	2	0.4	2 (100)	0 (0)	67.50
Takayasu's arteritis	4	0.8	4 (100)	0 (0)	41.50
Granulomatosis with polyangiitis	4	0.8	2 (50)	2 (50)	45.50
Eosinophilic granulomatosis with polyangiitis	1	0.2	0 (0)	1 (100)	16.00
Hypersensitivity vasculitis	7	1.4	3 (42.9)	4 (57.1)	25.57
IgA vasculitis	6	1.2	3 (50)	3 (50)	29.67
Behcet's disease	55	11.3	18 (32.7)	37 (67.3)	32.91
Infections and related arthritides					
Septic arthritis	5	1.0	2 (40)	3 (60)	41.80
Viral arthritis (hepatitis A virus)	5	1.0	2 (40)	3 (60)	24.20
Tuberculous arthritis	2	0.4	1 (100)	1 (100)	36.50
Rheumatic fever	1	0.2	1 (100)	0 (0)	11.00
Diffuse pain syndromes					
Fibromyalgia	7	1.4	6 (85.7)	1 (14.3)	34.86
Metabolic bone disease					
Osteomalcia	1	0.2	1 (100)	0 (0)	15.00
Osteoporosis	2	0.4	2 (100)	0 (0)	57.50
Nonrheumatic systemic disorders					
Osteogenesis imperfecta	2	0.4	1 (50)	1 (50)	16.50

Abbreviations: CREST, calcinosis, Raynaud's phenomenon, esophageal dysmotility, sclerodactyly, and telangiectasia; IBD, inflammatory bowel disease; IgA, immunoglobulin A.

#### Medical Staff

In northwestern Syria, where the population is 5.5 million, there are currently only three adult rheumatology specialists (0.07 rheumatologists per 100,000 people) registered with the health directorates. It is worth noting that two of them joined the region after 2021, which means that until the end of 2021, there was only one registered specialist in rheumatology. Furthermore, there is an unlicensed doctor who practices rheumatology and has not completed the required training to practice the profession. Additionally, there is currently no pediatric rheumatologist available in the area. As a result, many patients with Rheumatologic conditions seek treatment from orthopaedic or internal medicine specialists, who may lack the necessary expertise in this specialized field.

#### Rheumatology Medical Facilities

There is currently no hospital that is exclusively dedicated to the Department of Rheumatology. However, three hospitals have a rheumatology clinic. One of these clinics is supervised by an internal doctor, while the other two are supervised by rheumatologists, who have only recently—less than a year ago—joined the region.

#### Laboratory and Radiological Investigations

The conflict in northern Syria has had a severe impact on the availability of rheumatology investigations. Due to the destruction of health care facilities and restrictions on importing necessary equipment, many of these investigations, including specific tests for rheumatology conditions, are no longer available. This shortage has resulted in increased costs for some investigations, making them unaffordable for many patients. As a result, individuals with rheumatic disease face significant challenges in accessing the screenings they need, particularly due to financial limitations resulting from poverty.

Furthermore, the ongoing conflict has caused a deterioration in the quality of certain investigations. For instance, there is a lack of expertise among radiologists in interpreting magnetic resonance imaging (MRI) of the sacroiliac joint, which hampers the diagnosis of various diseases, especially axial spondyloarthropathy. Additionally, the absence of qualified radiologists capable of performing joint ultrasonography increases the demands on MRI.

Table 2 provides a summary of the diagnostic tests currently available for rheumatic disease in northwest Syria, along with information on the affordability of these tests for patients. However, it should be noted that accurately counting the number of tests is a challenge since many of them are conducted in private centers or sent to neighboring countries.

**Table 2:** The available rheumatic disease diagnostic test in northwest Syria and its affordability to the patients

Investigation	Availability	Cost
**General** b **lood** t **ests**		
**Erythrocyte** s **edimentation** r **ate (ESR)**	Available	Affordable
**C-reactive protein (CRP)**	Available	Affordable
**Uric acid**	Available	Affordable
**Complement (C4 and C3)**	Available	Affordable
**Cryoglobulins**	Not available	–
**Autoantibodies**		
**Rheumatoid** f **actor (RF)**	Available	Affordable
**Cyclic** c **itrullinated** p **rotein (CCP)**	Available	Nonaffordable
**Antinuclear factor**	Available	Nonaffordable
**Anti-double** **stranded DNA** ( **dsDNA** )	Available	Nonaffordable
**Anti-Smith**	Available	Nonaffordable
**Anti-Scl-70/anticentromere**	Available	Nonaffordable
**Anti-Jo-1 (antihistidyl tRNA synthetase)**	Available	Nonaffordable
**Anti-U1-RNP**	Not available	–
**Ant** i- **ribosomal P**	Not available	–
**Antihistone**	Not available	–
**Anti-Ro/SSA**	Available	Nonaffordable
**Anti-La/SSB**	Available	Nonaffordable
**Phospholipid antibodies**	Available	Nonaffordable
**Anti** n **eutrophil** c **ytoplasmic** a **ntibodies (ANCA)**	Available	Nonaffordable
**HLA typing**		
**HLA-B27**	Available	Nonaffordable
HLA-DRB1*04	Not available	–
**HLA-B5**	Available	Nonaffordable
**HLA-B52**	Not available	Nonaffordable
**Imaging**		
**Plain radiography**	Available	Affordable
**Computed** t **omography (CT) scan**	Available	Affordable
**Magnetic** r **esonance** i **maging (MRI)**	Available	Nonaffordable
**Ultrasonography**	Available	Affordable
**Isotope scanning (** t **echnetium-99m)**	Not available	–
**D** E **XA scan**	Not available	–
**Synovial fluid**		
**CBC**	Available	Affordable
**Culture**	Available	Affordable
**Crystals**	Not available	–
**Others**		
**Electromyography (EMG)**	Available	Nonaffordable
**Tissue** b **iopsy**		
**Lung, kidney, skin biopsy**	Not available	–
**Muscle biopsy**	Available	Nonaffordable

Abbreviations: CBC, complete blood count; DEXA, dual-energy X-ray absorptiometry; HLA, human leukocyte antigen.

#### Rheumatic Medications

The ongoing conflict in the region has resulted in a shortage of medical supplies, including generic rheumatology drugs. This is due to the destruction of pharmaceutical factories and restrictions on the import of these medicines. As a result of this shortage, the cost of medicines has increased, making them unaffordable for many patients. Regrettably, most medications for rheumatic diseases are unavailable. There are a few options available for rheumatology patients, but these are also costly. For instance, methotrexate, one of the most frequently prescribed medications for such conditions, comes at a high price. This poses a challenge for many patients living in poverty who depend on this medication to control their symptoms.

Moreover, the conflict has disrupted the supply chain of medicines, making it difficult for pharmaceutical companies to distribute their products to the region. Even if medicines are available, they may become unusable due to the lack of electricity to store these drugs in warehouses, especially biological drugs. This situation has had a significant impact on the health of the people in the region, who are struggling to access the medical care they need.

The unavailability of medications for rheumatic diseases can have severe consequences for patients suffering from such diseases. Many of these ailments require ongoing treatment with disease-modifying generic drugs and immunosuppressive drugs to control symptoms and prevent the disease from progressing. In the absence of these medications, patients may experience worsening of their symptoms, decreased quality of life, and an increased risk of complications associated with their condition and may resort to excessive use of steroids and analgesics (nonsteroidal anti-inflammatory drugs).

It is crucial to ensure that the medicines for rheumatic diseases are both available and affordable for patients in need. Table 3 provides a summary of the available medicines in northwest Syria along with the patients' ability to afford them. However, it should be noted that there is a challenge in accurately counting these medicines, as most of them are obtained from private pharmacies or purchased by patients from neighboring countries.

**Table 3:** The available medicines for rheumatic disease in northwest Syria and its affordability to the patients

Drugs	Availability	Cost
**Anti-inflammatory agents**		
**NSAIDs**	Available	Affordable
**Glucocorticoids**	Available	Affordable
**Colchicine**	Available	Affordable
**Analgesics**		
**Acetaminophen**	Available	Affordable
**Opiates**	Available	Nonaffordable
**Tramadol**	Available	Affordable
**Topical agents**	Available	Affordable
**Nonbiologic DMARDs**		
**Methotrexate**	Available	Nonaffordable
**Hydroxychloroquine**	Available	Nonaffordable
**Sulfasalazine**	Available	Nonaffordable
**Leflunomide**	Not available	**–**
**Azathioprine**	Available	Nonaffordable
**Cyclophosphamide**	Available	Nonaffordable
**Mycophenolate mofetil**	Available	Nonaffordable
**Cyclosporine**	Available	Nonaffordable
** Biologic DMARDs a**		
**TNF-** α **inhibitor**	Available	Nonaffordable
**Ustekinumab**	Not available	**–**
**Abatacept**	Not available	**–**
**Belimumab**	Not available	**–**
**Anakinra/canakinumab**	Not available	**–**
**Rituximab**	Available	Nonaffordable
**Tocilizumab**	Not available	**–**
**Tofacitinib**	Not available	**–**
**Urate-lowering therapy**		
**Allopurinol**	Available	Affordable
**Febuxostat**	Available	Affordable
**Probenecid**	Not available	**–**
**Pegloticase**	Not available	**–**

Abbreviations: DMARD, disease-modifying antirheumatic drug; NSAID, nonsteroidal anti-inflammatory drug; TNF, tumor necrosis factor.

a Storing and transportation conditions may affect the efficacy of these medications as the electric supply is continuously interrupted.

## Discussion

The shortage of rheumatologists in the workforce is a major problem worldwide, especially in developing countries such as those in the Middle East. According to the Arab League of Associations for Rheumatology (ArLAR) Research Group (ARCH), the average number of rheumatologists in Arab countries is only 0.84 per 100,000 people. 7 In the northern region of Syria, there are only 0.07 rheumatologists available for every 100,000 people. This scarcity is similar to that of certain other countries such as Nicaragua (0.07 per 100,000 people), Pakistan, Nigeria (0.01 per 100,000 people), and India (0.01 per 100,000 people). However, this number increases significantly in developed countries such as France (3.8 per 100,000), the United States (1.78 per 100,000), and the United Kingdom (0.84 per 100,000). 8

According to this research paper, the most commonly observed joint disease is rheumatoid arthritis, which contradicts the findings of other published papers in the medical literature that state that osteoarthritis is the most prevalent arthritis disease. 8 9 10 11 12 13 The reason for this discrepancy is that in our study, most osteoarthritis cases were treated by orthopaedic doctors. The average age of patients diagnosed with joint diseases corresponds to the age ranges reported in medical literature. Additionally, it has been observed that juvenile idiopathic arthritis is the most frequently occurring joint disease in the pediatric age group, followed by familial Mediterranean fever.

The prevalence of rheumatic diseases among males and females is consistent with most studies 9 10 11 13 ; however, it has been observed that lupus, rheumatoid arthritis, and other connective tissue diseases are more prevalent in females. It has been noted that gout and spondyloarthropathy are more common in males, 9 10 11 13 while Behcet's disease is more common in males in our study than in Istanbul, Turkey. 14 The study observed a rise in the number of cases of scleroderma, without any clear explanation. Further research is required to understand this trend. On the other hand, the study found that the number of rheumatic fever cases is lower than in other studies. This may be because most patients receive diagnosis and treatment from pediatricians, without being referred to rheumatology clinics.

The burden of musculoskeletal diseases increased significantly between 2000 and 2015. It is now the second leading cause of disability worldwide. 15 Rheumatic diseases, particularly inflammatory conditions, should not be considered solely in terms of their physical health effects. These diseases not only elevate the risk of organ dysfunction but also result in gradual disability and increased psychological burdens for those affected. As a result, the overall quality of life is reduced, and financial difficulties arise due to lower income and the increasing expenses of managing these conditions. 16 Early diagnosis and timely treatment are crucial for patients to maintain their professional and social engagements, minimize the adverse consequences of the disease, and preserve their overall well-being. Accordingly, prioritizing early detection and implementing appropriate therapies can help individuals remain active in their work and social lives and reduce the negative effects of rheumatic diseases.

The impact of war and displacement on disease activity and quality of life in patients with rheumatic diseases in northwestern Syria is a complicated and multifaceted issue that requires careful assessment. The conflict's socioeconomic impacts have also contributed to the deterioration of the quality of life of patients with rheumatic diseases. Many have lost their homes, their means of livelihood, and their social support systems, which can have a significant impact on their mental and physical health.

Depressed mood levels are significantly higher in rheumatoid arthritis, osteoarthritis, and fibromyalgia patients than in healthy controls, as shown by a systematic review and meta-analysis. 17 In a cross-sectional study, 20% of those with arthritis had an anxiety disorder, compared with 13% of those without arthritis. 18 One study on veterans revealed that 94.4% of the cases were attributable to injuries sustained during war. 20 There is growing evidence in the literature that posttraumatic stress disorder (PTSD), caused not only by war but also by other factors, may be linked to the development of autoimmune disorders. 21 Studies have reported a higher prevalence of myofascial pain and rheumatoid arthritis among U.S. military personnel with PTSD. 22 23 A cross-sectional study in Syria revealed high rates of mental health impact: 44% with likely severe mental disorders and 27% with both severe disorders and full PTSD. 19

In Syria, 35.2% of the population lives below the poverty line, which is defined as earning $3.65 or less per day, according to the World Bank. 24 In a recent poll conducted in northern Syria, over half of the surveyed population earned less than US$100, which is equivalent to less than $3.3 per day on average. 25

To help rheumatology patients living in conflict-affected areas, specific interventions are necessary. These interventions should aim to tackle the unique difficulties that these patients face. Examples of such interventions can include providing mobility aids to help patients manage their symptoms and creating community-based support networks that can provide ongoing psychosocial support.

## Challenges and Potential Solutions for the Development of Rheumatology in Northwest Syria

One of the main challenges in the field of rheumatology in northern Syria is the shortage of human resources to provide medical services, as well as the low number of trainers available. These concerning statistics could have a negative impact on the quality of care for rheumatology patients, potentially leading to increased morbidity and mortality rates. Table 4 outlines the major challenges faced in the field, along with proposed solutions.

**Table 4:** Challenges and potential solutions for the development of rheumatology in northwest Syria

**Lack of human resources**	• **Training of internists on the specialization of rheumatology** • ** Launching a training program for resident doctors in rheumatology through the SBOMS 26**
**Migration of** r **heumatologist**	• Providing extra salaries or incentives to retain rheumatologists currently working in northwest Syria
**Lack of training programs**	• Launching a fellowship program in rheumatology in collaboration with expatriate doctors • Activating periodic medical missions for expatriate rheumatologist to northern Syria • International missions in the field of rheumatology for trainee doctors to neighboring countries, with the stipulation of their return to northern Syria after the end of training
**The high cost and the unavailability of most rheumatic investigation**	• Encouraging governmental medical authorities and nongovernmental medical organizations to provide these investigations in northern Syria
**Many people do not know that the specialization of rheumatology is independent of the specialization of orthopaedics**	• Educating patients in northern Syria about the importance of this specialty in treatment • Encouraging government medical authorities to refer rheumatology cases to specialized doctors through the referral system

Abbreviation: SBOMS, Syrian Board for Medical Specialties.

## SBOMS: Syrian Board of Medical Specialties

The Syrian Board for Medical Specialties (SBOMS) 26 is an academic institution that focuses on training resident doctors in northwestern Syria. The training takes place in accredited hospitals, and upon completion, doctors receive a certificate of specialization. The SBOMS program aims to deliver excellent training to resident doctors in all specialties by specialist doctors and supervisors located in northern Syria. To address the lack of availability or rarity of certain specialties, high-quality distance education is utilized. The training was conducted with the guidance of Syrian doctors who are currently residing outside their home country. In the field of rheumatology, specialized lectures are conducted regularly, according to the latest recommendations and scientific methodology. These lectures aim to fill the gaps in general topics in the specialization. Additionally, there is a weekly rheumatology clinic, which many residents attend periodically. For complex consultations, there is online communication available with expatriate rheumatologists from Qatar, the United States, and Saudi Arabia. This situation highlights the need to train new local rheumatology doctors to address the shortage of health care personnel in this field.

At the time of writing this article, SBOMS currently does not offer a specialty in rheumatology. However, the organization hopes to collaborate with interested partners in education to open this specialty soon. By highlighting the significance and challenges associated with this field, SBOMS aims to raise awareness and encourage the development of the rheumatology specialty.

## Conclusions

There is a severe shortage of rheumatologists and medications for rheumatic diseases in northwestern Syria, despite the high prevalence of such diseases in the region. Due to the impact of war and displacement on patients with rheumatic diseases in northern Syria, addressing the issue requires a multifaceted approach. The solution involves breaking down barriers to health care and medication access, providing human resources, supporting patients' psychosocial needs, and conducting targeted research. This study aims to overview the challenges facing this specialization in a war-torn country and identify critical gaps that need addressing to mitigate their impacts.
